# Intrathecal Ziconotide Long‐Term Management in Chronic Postamputation Pain

**DOI:** 10.1155/cria/9977830

**Published:** 2026-08-02

**Authors:** Rafael Galvez Mateos, Nicolás Cordero Tous, Majed Katati, Beatriz Lechuga Carrasco, Manuel A. Sánchez García

**Affiliations:** ^1^ Pain Unit, Hospital Virgen de las Nieves, Granada, Spain, hvn.es; ^2^ Neurosurgery Service, Hospital Virgen de las Nieves, Granada, Spain, hvn.es

**Keywords:** analgesic control, chronic pain, intrathecal ziconotide, long-term, residual limb pain

## Abstract

The clinical case of a young patient with intractable residual limb pain is presented. Following the initial administration of opioids and subsequent overuse, the patient was switched to intrathecal (IT) ziconotide infusion with complete opioid withdrawal. After 15 years of treatment with ziconotide infusion, the patient achieved sustained analgesic control and excellent functional recovery. IT ziconotide showed good long‐term tolerability and safety.

## 1. Introduction

Postamputation pain, referred to as persistent residual limb pain, is characterized by mixed nociceptive and neuropathic components that persist in the surgical residual limb for more than 3 months. It is often of high intensity and difficult to treat [1]. While most patients experience resolution of residual limb pain within the first few months after amputation, a minority (10%–23%) continue to experience chronic pain, which can impact their quality of life [1–5].

The main predisposing factors include intense pain prior to amputation, local infection (with or without osteomyelitis), aggressive surgery with extensive tissue destruction, female gender, genetic factors, and the development of neuromas [4]. Psychological factors such as anxiety, depression, or catastrophizing are also of major relevance [2, 5]. Pain may occur spontaneously or be triggered by prosthesis contact, resulting in greater functional limitations. It is often accompanied by sensations of pressure, tightness, burning, or pain from rubbing [3, 6].

Residual limb pain should not be confused with phantom limb pain, which occurs in the amputated area. The latter initially affects up to 85% of patients and usually diminishes over the course of months, being more intense after proximal amputations or in cases with severe preoperative pain. Occasionally, phantom pain may occur concurrently with residual limb pain [6].

Residual limb pain is localized and clearly identifiable, which allows different treatment alternatives. The analgesic strategy emphasizes the importance of aggressively treating preoperative pain and minimizing it as much as possible, as well as immediately treating postoperative pain, because these are directly associated with the development and intensity of chronic residual pain [4]. Intraoperative locoregional anesthesia and postoperative epidural analgesia appear to be effective in reducing its incidence and severity [4].

Nonpharmacologic interventions include massage, physical therapy, and other physical rehabilitation techniques. Emotional support is also essential following amputation in most cases [3, 7, 8]. Interventional measures are recommended from the initial diagnosis of the residual limb pain. Ultrasound‐guided local nerve blocks can be very useful. Posterior radiofrequency of the nerves can also be a highly effective analgesic technique [9]. Neuromodulation techniques, such as spinal cord stimulation (either tonic or high‐frequency), have shown great interest and are increasingly supported by several publications. Spinal cord stimulation, whether tonic or high frequency, is one of the most effective analgesic alternatives for resistant pain in the residual limb in recent years, and an increasing number of published works endorse the technique [7, 8, 10].

Pharmacologic treatment ideally targets the underlying cause of residual limb pain, which is difficult in most cases. Analgesic options include anticonvulsants (such as gabapentin or pregabalin), some antidepressants (such as tricyclics) and opioids for severe refractory cases [7, 10, 11]. Intravenous ketamine has been reported to provide pain relief in some cases, and high‐concentration (8%) capsaicin patches have been shown to reduce chronic residual limb pain [12].

Intrathecal (IT) spinal infusion of analgesics is considered the fourth step of the analgesic ladder and is reserved for patients with severe, complex, refractory chronic pain. This technique addresses one of the most challenging problems faced by patients and professionals: refractory chronic pain [13, 14].

Morphine was the first opioid used for spinal infusion, and it remains the most widely used opioid. Its use in treating chronic refractory pain of both oncologic and nononcologic origin is supported by the largest body of published research. Based on administration of the opioid at the subarachnoid level, close to the central nervous system, very low doses of morphine are required to achieve a strong analgesic effect [14].

Ziconotide is a potent synthetic nonopioid analgesic, derived from the venom of the marine snail *Conus magus*. It acts as a potent analgesic and is administered exclusively intrathecally. Its mechanism of action involves blocking voltage‐gated N‐type calcium channels [14, 15].

Here, we present a case of a young woman with residual limb pain refractory to opioids, who has been successfully treated with long‐term IT ziconotide.

## 2. Case Presentation

A 29‐year‐old female patient with no previous history of interest, who suffered a traffic accident with crush injury to the right leg and fracture of the right tibia, was treated with osteosynthesis. She subsequently developed an infection and underwent an infracondylar and then supracondylar amputation.

She suffered from severe residual limb pain and right thigh pain with initial clinical phantom limb syndrome, and analgesic treatments were ineffective. Previous treatments included the use of different antidepressants and antiepileptics, as well as the use of multiple opioids, which were gradually added over time. The patient was a frequent visitor to the emergency department and visited several specialists for her condition.

### 2.1. Initial Presentation and Analgesic Regimen Upon Arrival

Upon presentation to the pain unit, the patient exhibited severe continuous neuropathic pain in the residual limb, with a visual analog scale (VAS) rating of 7, which increased to VAS 9 on mobilization or rubbing, and a Karnofsky index score of 70. She initially presented with phantom limb syndrome, but then a residual limb pain remained, and she was unable to place the affected limb on the pavement and was unable to wear an orthosis due to the presence of pain. She spent most of her time in a seated position and required the use of a wheelchair for mobility. She presented with a high emotional component, with severe impairment, and also presented a high level of additive component with overuse of opioids.

Analgesia on arrival. The patient was referred to the pain unit 8 years after the onset of postamputation pain, with a history of high intake of oral and transdermal opioids and other analgesics: fentanyl 100 μg patch/72 h, MST 30 mg/8h (controlled‐release morphine) and transmucosal fentanyl 800 μg on demand (2–6 times daily), pregabalin 150 mg/12 h, duloxetine 30 mg/12 h, and paracetamol and metamizole on demand.

### 2.2. Initiation of IT Ziconotide Therapy and Long‐Term Follow‐Up

After evaluation of the patient, ziconotide spinal infusion was chosen as an alternative due to the high neuropathic and nociceptive pain component and opioid overuse (Table 1). A subarachnoid catheter and an implantable variable flow pump were placed, and spinal infusion of ziconotide was started at a dose of 1 μg/day, with progressive withdrawal of opioids. The dose of ziconotide was increased to 2.2 μg after 15 days, and to 4 μg after 30 days, providing significant relief to the patient, and further increased to 6.2 μg after 2 months, achieving objective relief from residual limb pain and reduction in hyperalgesia and allodynia, enabling the patient to touch the affected area, and allowing a marked reduction in the need for opioids until almost their elimination. The phantom limb syndrome was reduced over time. After 3 months, the dose was increased to 7.2 μg, but the patient experienced headaches and vertigo of mild intensity, so ziconotide was tapered back to 6.2 μg and the symptoms disappeared. Pain relief was maintained, and the dose was subsequently increased to 7.2 μg, and after 4 years, to 8 μg, maintaining this dose for 2 years. The dose was tapered back to 7.2 μg over the years.

**TABLE 1:** Timeline of intrathecal ziconotide therapy and clinical outcomes over 15 years.

Time since IT ziconotide initiation	Intervention/dose	Clinical events & outcomes	Adverse events/actions
Baseline (Month 0)	IT ziconotide started at 1 μg/day (implantable variable flow pump)	Progressive withdrawal of all opioids. Severe residual limb pain (VAS 9). Karnofsky 70	—
2 weeks	Dose increased to 2.2 μg/day	Initial pain relief reported	—
1 month	Dose increased to 4 μg/day	Further improvement; decreased hyperalgesia and allodynia	—
2 months	Dose increased to 6.2 μg/day	Marked pain relief; almost complete opioid discontinuation. Improved limb contact tolerance	—
3 months	Dose increased to 7.2 μg/day	Excellent analgesia and functional recovery	Mild headache and vertigo (solved after reducing the dose back to 6.2 μg/day)
4 years	Dose increased to 8 μg/day	Stable analgesia (VAS 2–3)	—
6 years	Replacement of pump to fixed‐flow model	Maintained pain control	—
11 years	Replacement of spinal catheter due to semiobstruction	Maintained therapy without complications	Mild local discomfort at insertion site
15 years (current)	Dose maintained at 7.2 μg/day	Stable analgesia (VAS 2‐3). No opioids required. ECOG 0, Karnofsky 90. Full functional recovery. Working as a clinical assistant	Mild local discomfort; no systemic adverse effects

*Note:* IT, intrathecal.

Abbreviations: ECOG, Eastern Cooperative Oncology Group; VAS, visual analog scale.

After 6 years, the pump was replaced with a fixed‐flow pump, and after 11 years, the spinal catheter was replaced due to semiobstruction of the catheter. The patient reported some local discomfort at the site of lumbar catheter insertion. The first catheter tip was placed in the thoracic spine between T9 and T10 and the second one in between T10 and T11.

The patient has been on IT ziconotide for 15 years, at a dose of 7.2 μg for the last 4 years. Analgesia with pregabalin is maintained at a dose of 150 mg/12 h, with dexketoprofen as rescue medication. Pain is currently stable with VAS 2‐3, and her quality of life is good, with an Eastern Cooperative Oncology Group (ECOG) performance status of 0 and a Karnofsky index score of 90. The patient wears an orthosis for ambulation. Treatment with ziconotide enabled the patient to complete her primary and secondary education and to be currently working as a clinical assistant.

IT ziconotide was prescribed and administered as part of routine clinical practice at our institution. The medication was obtained through the hospital pharmacy according to standard institutional procedures.

## 3. Discussion

Ziconotide was approved by the FDA in 2004 and by the EMA in 2005 for the treatment of refractory chronic pain and is the only nonopioid analgesic approved by the FDA for spinal analgesia. Among analgesics approved for IT administration in refractory pain, ziconotide is recommended in the 2017 Polyanalgesic Consensus Conference (PACC) spinal infusion guidelines. This positions ziconotide as a first‐line drug for treating both nociceptive and neuropathic pain, as well as oncologic and chronic benign pain, whether diffuse or localized [14]. A recent comprehensive review highlighted the favorable benefit–risk profile of ziconotide when titrated cautiously and under close monitoring [16].

The present case is one of the few reports describing long‐term IT ziconotide treatment and, to our knowledge, the first case of residual limb pain managed successfully with IT ziconotide as monotherapy.

The patient responded rapidly to IT ziconotide treatment, achieving significant pain relief at a dose of 6.2 μg after approximately 2 months. The patient described this outcome as highly favorable to her well‐being, with reduced pain and local hyperalgesia in the residual limb. The patient’s good quality of life is reflected in an ECOG of 0 and Karnofsky index of 90.

Regarding adverse events, the patient reported a few mild and transient adverse events related to ziconotide that disappeared when the dose was reduced.

With regard to safety and tolerability, the patient initially reported a few drug‐related adverse events, such as headache and vertigo, after 2 months with a dose of 6.2 μg. These events were mild and transient and resolved after reducing the dose. No other systemic effects have been detected over the years, apart from increased pain when reloading has been delayed. The remaining adverse events reported by the patient were related to some local discomfort at the lumbar catheter insertion site and catheter replacement due to semiobstruction of the catheter.

Other common adverse effects reported in the literature include peripheral edema, diarrhea, constipation, or somnolence. Studies indicate that ziconotide is associated with lower rates of constipation and vomiting compared to morphine [17]. More serious adverse events, such as hallucinations, delirium, and psychotic episodes, have been reported but did not occur in this patient, possibly due of the slow and cautious dose escalation, with the exception of the first four doses (initially 1 μg, followed by an increase to 2.2 μg/day after 15 days, 4 μg after 30 days, and 6 μg after 2 months). All subsequent increases were in increments of 0.5 μg of ziconotide. Nevertheless, the new PAAC 2024 guidelines recommend increments of only 0.5 μg/day for titration used [14, 18–21]. It should also be noted that the IT route allows for lower systemic exposure, thereby reducing systemic adverse events [22].

Despite the long‐standing use of IT therapy in chronic pain, few long‐term controlled trials have been conducted and published, and evidence remains limited [18–21, 23, 24]. The challenge is even greater and more complicated when we refer to IT ziconotide, where experience is less extensive than with morphine, and its approval and market launch occurred after morphine [19, 25]. Although current data support a favorable benefit–risk ratio when used as a monotherapy, information on long‐term real‐world outcomes remains scarce [16].

IT analgesic infusion is a valid option for chronic refractory pain, but it requires appropriate patient selection, shared care, and interdisciplinary coordination among professionals. Specialized nursing care, as well as close follow‐up and monitoring of patients, is also necessary.

However, there is a lack of controlled studies with clinical evidence, as well as observational studies, on the long‐term efficacy and safety of IT ziconotide. This is important because ziconotide is used to treat chronic pain, which could last for years or be permanent.

The case report presented here demonstrates the potential of IT ziconotide to provide stable and durable pain relief over a very long treatment period, with sustained efficacy and favorable tolerability. This experience supports its role as a valuable therapeutic option in selected patients with chronic refractory neuropathic pain, such as residual limb pain.

## 4. Conclusions

IT infusion of ziconotide has improved and maintained continuous pain relief for 15 years, and the patient has achieved high analgesic control and excellent functionality. Also, IT ziconotide showed long‐term good tolerance and safety. IT ziconotide may represent an analgesic option for residual limb pain with an uncontrollable neuropathic component, relieving pain, hyperalgesia, and allodynia, while improving comorbid symptoms and patient autonomy.

## Author Contributions

Rafael Galvez Mateos: conceptualization, data curation, formal analysis, investigation and methodology, resources, supervision, and writing–review and editing.

Nicolás Cordero Tous: recruitment and data collection of the patient, data curation, formal analysis, investigation and methodology, resources, supervision, and writing–review and editing.

Majed Katati: formal analysis, investigation, and methodology.

Beatriz Lechuga Carrasco: recruitment and data collection of the patient, data curation, formal analysis, investigation, and methodology.

Manuel A. Sánchez García: recruitment and data collection of the patient, data curation, formal analysis, investigation, methodology, resources, supervision, and writing–review and editing.

## Funding

Esteve Pharmaceuticals SA provided an unrestricted grant to support the preparation and publication of this case report.

## Disclosure

All authors have read and agreed to the published version of the manuscript. Esteve Pharmaceuticals had no role in the clinical management of the patient, interpretation of the case, or in the decision to publish the case report. No study drug or clinical material was supplied by Esteve Pharmaceuticals. The authors retained full control over the content and final approval of the manuscript. The financial support provided by Esteve Pharmaceuticals SA was in the form of an unrestricted grant to support medical writing assistance and publication‐related activities and did not influence the content of the manuscript.

## Consent

Written informed consent was obtained from the patient for the publication of this case report. The patient was informed that no identifiable personal information would be disclosed.

## Conflicts of Interest

The authors declare no conflicts of interest.

## Data Availability

All basic clinical data have been reported. Data are available on reasonable request by contacting the corresponding author.
